# *“It is meaningful; I feel that I can make a difference” -*A qualitative study about GPs’ experiences of work at nursing homes in Sweden

**DOI:** 10.1186/s12875-015-0326-6

**Published:** 2015-08-28

**Authors:** Beata Borgström Bolmsjö, Eva Lena Strandberg, Patrik Midlöv, Annika Brorsson

**Affiliations:** Center for Primary Health Care Research, Department of Clinical Sciences in Malmö, Lund University, Jan Waldenströms gata 35, SE 205 02 Malmö, Sweden; Blekinge Centre of Competence, Karlskrona, Sweden

## Abstract

**Background:**

Swedish nursing homes (NH) have limited capacity. As a result elderly people living in NH represent the part of the elderly population in most need of care. In Sweden a General Practitioner (GP) is usually responsible for the medical care of all subjects living in a NH*.* The residents in NH seldom have adequate pharmacological treatment according to diagnosis and often have polypharmacy and/or inappropriate medical treatment regarding concerns of declining renal function. What prevents optimal care for the elderly is multifaceted, but there is limited research on how GPs experience their work with the elderly in NH in Sweden.

This study aims to illuminate the GPs’ work with the elderly in NH to provide input on how the care can be improved, as well as to identify potential obstacles for good quality of care.

**Methods:**

This qualitative study is based on individual semi-structured interviews with 12 GPs and a follow-up focus group discussion with six of the interviewed GPs.

The interviews were analysed with systematic text condensation, with the process leading to identify categories and themes. Thereafter, the themes were discussed among six of the participating GPs in a focus group interview.

**Results:**

Two main themes were identified: concern for the patient and sustainable working conditions. The principal focus for the GPs was to contribute to the best possible quality of life for the patients. The GPs described discordance between the demand from staff for medications and the patients’ actual need of care. GPs found their work with NH enjoyable. Even though the patients at the NH often suffered from multiple illnesses, which could lead to difficult decisions being made, the doctors felt confident in their role by having a holistic view of the patient in tandem with reliable support from the nurse at the NH.

**Conclusion:**

Working with NH patients was considered important and meaningful, with the GPs striving for the patient’s well-being with special consideration to the continuum of ageing. A continuous and well-functioning relationship between the GP and the nurse was crucial for the patients´ well-being.

## Background

Together with the increase in the ageing population there is now a greater demand for a primary health care with well-functioning means for taking care of the elderly [1, 2]. Nursing Homes (NH) serve as long term care facilities for frail elderly in many countries, including Sweden. The quantity and quality of NH differs between, as well as within, countries and there are large differences concerning the contribution of physicians and nurses [3]. An international survey from 2013 showed that about one third of NH around the world have physicians paying regular visits. This survey also confirmed that the residents in NH are multimorbid and frail, with 82 % of the residents taking six or more medications a day [4]. Nursing homes (NH) in Sweden have a limited capacity and only 5.2 % of Swedish residents aged 65 and over live permanently in a NH [5]. Hence, elderly people living in NH in Sweden represent the part of the elderly population that are most frail and in greatest need of care [6]. As NH residents are a vulnerable group with multi-complex needs; difficult medical decisions have to be considered, along with dignity conserving aspects [7].

In Sweden, medical care for those living in NH is managed by the primary health care centre (PHCC). In most cases a General Practitioner (GP) takes care of all NH residents and pays weekly visits. The GP meets with the nurse and they have a discussion about the patients. In addition the nurse will have identified patients that are in need of a medical assessment. Usually, the GP is contacted by phone or fax in between the weekly visits for more acute consultations. During the rest of the week, the GP works with outpatients at the PHCC.

Research from NH has shown that the subjects seldom have adequate pharmacological treatment according to diagnosis and often have polypharmacy and/or inappropriate medical treatment in concerns of declining renal function [8, 9]. One reason for this is suggested to be due to a lack of knowledge [10] and GPs have expressed a need for clear information on the benefit/risk ratio of preventive medication in the very old and frail [11]. Other explanations are lack of time and insufficient economic resources [12, 13]. Extensive curative/preventive drug therapy and late shift to palliative care has been shown and indicates prognostic difficulties in NH patients often with dementia and multimorbidity [14]. By attempting to give dignity-conserving care NH physicians and nurses often experience conflicting values in daily care and barriers caused by a lack of resources [7]. Research from the UK, where one NH may have many different GPs because of the tradition of keeping the same personal GP over the years, showed that regular medical rounds of the doctor was preferred by NH managers but was increasingly being replaced by visits on request due to the GPs’ increasing workload [15].

Also, decisions regarding the hospital admission of NH residents and decisions on palliative care approach may present a difficult dilemma for the GP. There is a need for further research for strategies to optimise and to possibly avoid inappropriate admissions [16].

A previous Swedish qualitative study on this field illustrates the problems with inappropriate hospital admissions of NH residents [17], but the focus has mainly been on the experience of the nurses. The GP´s experience of the work with NH in Sweden had not been studied prior to the current study being carried out.

Sweden has a shortfall of GPs in PHC, as is the case in many other countries around the world. A large proportion of the GPs will retire in the coming years and there are not enough qualified young GPs to replace them. As a result this will lead to a great challenge for the future of PHC in Sweden. Hence, there is a need for coping strategies [18] for the remaining GPs to manage their work situation. The GPs need to approach a salutogenic perspective [19] and focus on factors that support their own health and well-being to create a sustainable PHC.

Even though the systems of elderly care differ between countries there is a major need for in-depth research around the work force and quality of care in NH to recognise opportunities for strategic improvement and to highlight priorities for education [4]. As the doctor-nurse relationship in health care institutions is very important for the efficiency of the system [20], it is of great interest to shed light on this link also in NH.

This study was performed to illustrate GPs’ experiences of the work with elderly living in NH in Sweden, to get input on how elderly care can be improved and to identify obstacles for good quality of care.

## Methods

This qualitative study is based on individual semi-structured interviews and a focus group discussion. The research question was how the GP experiences their work at nursing homes.

The point of departure consists of concrete descriptions of experienced events from the perspective of everyday life by the participants. The result is then a description of underlying comprehension of the experience, interpreted for assessing knowledge that could be significant for a wider community [21].

The text was analysed with qualitative content analysis through systematic text condensation [22]. We chose an editing style analysis [23] that was well suited for development of new concepts.

### Participants

We used purposive sampling by identifying GPs with varying NH experiences. The only inclusion criterion was that the GP was working in a NH and wanted to participate in the study. There were no exclusion criteria. In total 12 GPs participated, three men and nine women. They had been working in Primary Health Care (PHC) for between two and 38 years. The GPs worked at NH situated in different cities as well as in smaller towns and villages in the southern part of Sweden. All GPs made weekly visits to the NH and had responsibility for between 24 and 100 patients each.

### Semi-structured interviews

We developed an interview guide according to Kvale [24]. This emanated from the aim regarding how the GPs experience their work with the elderly. Three main areas with accompanying research questions were stated as following; 1) Describe the work at the NH. 2) How is the work at the NH valued and appreciated? 3) What is the objective of your work at the NH? These areas originated from the clinical practice and have been problematised in previous studies [13, 15]. Interview questions were developed from these research questions. The interview questions were short, simple and open to encourage the discourse.

The interviews were situated at a place that the GP felt was most convenient. Seven of the interviews were conducted at the PHCC where the GP worked. Three interviews were conducted at the research centre where the first author works and in one case in the first author’s home, and in another case at the home of the interviewee. The interviews typically lasted for about 35–40 minutes.

The interviews were recorded digitally, thereafter transcribed verbatim by the first author and a research assistant.

### Focus group

Two main themes were derived when analysing the interviews. To deepen these themes a focus group discussion with the interviewed participants was held. All of the 12 GPs were invited to the focus group discussion. Ten of the GPs were interested in participating but on the day of the meeting four of them reported well-founded reasons for absence (other work commitments, maternity leave and illness). A total of six GPs, three men and three women, with different lengths of experience participated in the focus group meeting.

The focus group discussion was held at the research centre where the authors work and lasted for around 90 min with a short break. The discussion was moderated by one of the co-authors as she had prior experience of moderating focus groups and is not a GP, which was thought could give more depth to the discussion. The first author assisted the moderator and took notes during the discussion to recall impressions during the conversation. The moderator based the discussion on the themes derived from the analysis of the interviews and used open-ended questions, thus allowing the participants to talk freely about the topic.

The focus group discussion was transcribed verbatim by a research assistant.

### Analysis

The analysis was performed stepwise according to Malterud [22]. First the text was read through several times in order to get to know the content. Thereafter, preliminary themes were derived from the interviews and through systematic text condensation meaning units connected with the preliminary themes were identified. The meaning units were condensed while preserving the essence of the meaning unit and then labelled with a code.

The codes were carefully sorted into subcategories and further into categories with internal homogeneity. The codes and categories were thought through and discussed among the co-researchers, and themes were derived from the manifest meaning of the content. An example of the text condensation in meaning units is shown in Table 1. The text from the focus group discussion was analysed similarly, although the codes were matched to the pre-existing categories and themes.

**Table 1:** Example of text condensation and coding

Meaning unit	Condensed meaning unit	Coding	Category
GP8:”Many medications, antibiotics and the like are given (to the patients) which they receive instead of the nursing care they actually need.”	Medicines are used instead of nursing care	Medicalisation	Care needs and medicalisation
GP11:”There is a focus on the doctor. And as I have very little chance to help the patient because what the patient actually is in need of is basic care needs, and now when he (the patient) feels bad…well… if one as a doctor then becomes “help needed” (as wanting to help) as someone says, a patient can get very many medications”	A service-minded doctor may prescribe too much medicines	Medicines vs. basic needs	
GP1:”No but it means that I sometimes have to compromise with what I really believe in…So it feels like it is a negotiation from all sides. So that they know what position I have and I know what position they have. Where I know that they are liberal with antibiotics, but you have to…, well I don´t want to say that I am a realist and not able to do what I want, but I am more careful about saying yes and no to things, and instead think about their (the nursing staff’s) preconditions.”	The doctor needs to be careful in the dialogue about ordinations	Negotiations about medicines	

### Ethical considerations

The study protocol was approved by the Regional Ethics Review Board at Lund University (case Number: Dnr 2014/219).

## Results

The picture that emerges from the interviews is that the main concern expressed by GPs who work at NH was the well-being of the patients. The nurse at the NH was seen as a key person for the patients at the NH and served as a mediator, negotiator and coordinator who was striving for their well-being. The GPs described discordance between the demand from staff for medications and the patients’ actual need of nursing care. The doctors wanted more nursing care and the nursing staff wanted more medications. This paradox was most evident for the GPs when encountering the powerlessness of not being able to fulfil the existential needs of the NH patients. 

The GPs found their work with NH enjoyable. It seemed to be a pleasant variation to everyday tasks at the PHCC and gave much-needed freedom to decide their own time schedule. Despite the fact that the patients at the NH suffered from multiple illnesses, which could lead to difficult decisions being made, the doctors felt confident in the role by having a holistic view of the patient combined with reliable support from the nurse at the NH. Two main themes were identified in the process of analysing the interviews: concern for the patient and sustainable working conditions. These themes contained three and four categories respectively (Table 2).

**Table 2:** Categories and themes

Categories	Themes
The continuum of ageing	Concern for the patient
Care needs and medicalisation	
The nurse as a key person for the well-being of the patient	
Holism	Sustainable working conditions
Collaboration	
Freedom and variation	
Meaningfulness	

The focus group discussion added some deeper understanding to the pre-existing categories and themes. However, no new categories or themes emerged from the analysis of the focus group discussion.

### Concern for the patient

The GPs were all careful to describe their concern for the patient based on the prevailing circumstances of caring for an older person during the end of life stage.

*“I want to give the patients a good quality of life, and I follow them in the continuum of ageing, with their progressive weakness and adapt medical interventions for this.”*(GP 10)

The GPs strove for less pain, less anguish and less loneliness for the patients to achieve the best possible well-being, often with help from the nurse. The GPs had different coping strategies to manage their concern for the patient.

The first category included in this theme was: *The continuum of ageing* (Table 3). The GPs were careful to explain the different perspective they took when working with the elderly at NH.

*”…the first priority is definitely to reduce suffering, reduce anxiety…try to make life meaningful for the patient. Diseases are secondary”* (GP4)

**Table 3:** Categories and examples of quotations for the theme “Concern for the patient”

Category	Quotations
The continuum of ageing	GP2:”Because it is a group of patients that are vulnerable, multimorbid, eh where you not always can diagnose the exact condition. So you must adapt to the situation. Emm, and they are so old that you don’t have to push the investigation in infinity, so you have to limit or constrain yourself. In that way it is challenging.”
Care needs and medicalisation	GP10:”And where it can be hard to gain support for examinations and follow-ups and help with observations and so… they like to call for sedatives, when it instead there is a need of attendance and other measures than medications.”
The nurse as a key person for the well-being of the patient	GP11:” well, I cooperate with the nurse named XX, and she is very good and that makes my work much easier and more pleasant. We can have a good dialogue and she… I feel that she has a good clinical sense and good intentions for the patient’s well-being.”

The focus was not to cure diseases but to give the best possible quality of life to the patient during the latter stages of life. This way of taking care of the patients could often be interfered with by relatives, who had other opinions, but the GPs felt confident in their perspective. Furthermore, the GPs usually could continue with their focus on well-being when communicating with the relatives about their anxiety regarding end of life care. There seems to be a large variation in how the GPs approach medications for elderly patients since guidelines around withdrawal of medications, and medications in general for the elderly, were perceived to be largely absent.

The second category was: *The care needs and medicalisation*. The GPs were careful to note that they wanted the patients to have as few medications as possible as they believed that many medicines had exhausted their impact when the patients were this elderly. The doctor´s part in deprescribing medicines along with highlighting the need of nursing care was often hard to manage. The GPs were frustrated around the care needs of the patients, which they thought could not be replaced by medicines.

“*It is not optimal that a patient gets a sedative drug instead of someone that holds her hand, but it is as good as it can get because there is no other way. That is frustrating of course, and sad, that I can’t influence this in any way” (GP 2)*

The GPs’ opinions were that the staff at the NH often asked for medicines to solve problems that were not in fact medical. Here a paradox was found as the employees at the NH were demanding more medicines and the GPs were demanding more nursing care. There was a need for ingenuity and for the GPs to be focused on solutions. The GPs could see that resources were lacking for nursing care and felt that the problem often was on a different level in the organisation, where the doctor had no influence. The conflict around medications was often managed by the nurse which leads into the third category in this theme: *The nurse is a key person for the patients’ well-being.* The GPs ability to provide well-being for the patients at the NH was described as being totally dependent on the nurse at the NH.

*”and I notice how doubtful I get of my own profession when not knowing the nurse. It is hard to know if she calls for too much action or not…”* (GP1)

If the nurse was confident in the role the GPs felt that they could provide the patients with good quality of care.

*“for everything to work optimally it is necessary that the nurse knows the patient, she is the key person”* (GP7)

A continuous relationship between the nurse and the GP rendered productive discussions on patients’ needs, as well as good communication strategies without irrelevant disturbances during the week. A confident nurse, with a thorough knowledge about the patients, could in many cases help the doctors to avoid unnecessary and sometimes harmful referrals to hospital. The nurse was also seen as a messenger for important information about the patients, as well as wishes and demands from the other staff at the NH and from the patients’ relatives. Sometimes this role of the nurse had to be more as a mediator when opinions about the patients’ needs diverged. The picture of the nurse was also as a coordinator regarding the patient and the NH. With well-prepared wards and well-functioning routines the GPs felt they could do an efficient job focusing on the well-being of the patient.

### Sustainable working conditions

The GPs found their work at NH enjoyable. The work was seen as important and meaningful for the GPs as they felt that they could make a difference for the NH patients. In addition, the work also gave a nice variation to everyday tasks at the PHCC. The commitment at NH provided a salutogenic work situation for the GPs.

The first category was (Table 4): *Holism*. The GPs felt that they had a much better overall picture of the patients at the NH, compared to when they met an elderly patient at the PHCC.

*“…so I think I can make more correct decisions at the NH, when I meet the patient in that environment”* (GP2)

**Table 4:** Categories and examples of quotations for the theme “Sustainable working conditions”

Category	Quotation
Holism	GP5:”Yes you have more time (at the nursing home) and … and well, you can’t compare it. Here (at the primary health care centre) old people come without any relative, sometimes with a nursing assistant that never has met them and you know… it is much easier to get a picture of the patient (at the nursing home)… but of course, they are also much sicker at the NH.”
Collaboration	GP7:”Yes, there (at the primary health care centre) you are alone in a different way and it is nice to have company… you can miss that at the primary health care centre, these wards where you sit down and discuss and talk with other professions, the nurses and so..”
Freedom and variation	GP12:”Very pleasant and it is sort of a relief compared to the ordinary work at the primary health care centre, you get away from the primary health care centre for a while every week and it is freer time, not the scheduled appointments all the time, but you go there and, sometimes you visit the patients and sometimes you just discuss the patients. It is more free and a different way of working with patients.”
Meaningfulness	GP7:” I believe and I think that it is extremely important and I think that one can contribute extremely (to the well-being of the patient) in many ways”

The doctors had regular meetings with their patients. They got thorough information about them from the nurse, staff and relatives and were provided with a careful list regarding medications. This provided a holistic view of the patients which helped the GP to make better medical decisions, and with more informed decisions the doctor was more satisfied. When the GP felt they had a good overall picture of the patient it was easier to avoid unnecessary actions and instead await and follow-up the condition.

The second category on GP satisfaction was: *Collaboration.* The GPs felt that the work at the PHCC was very lonely as due to stress it was often not possible to see any colleagues at all. Their work at the NH differed in that way.

*“the thing with taking to the nurses, talking to the relatives, you work together, that is actually quite enjoyable…”* (GP8)

At the NH the GP sat down with the nurse and the nursing staff to discuss the patients. This created a less lonely situation for the GP, which was appreciated. Even though the GPs appreciated the collaboration with the nurse at the NH they were all united in that the need for collegial discussions with other GPs involved in NH was greatly absent, but would be fruitful for their work. The GPs learnt more by doing as they had not had previous schooling or introduction by other colleagues to the work at the NH. In the interviews, particularly during the focus group discussion, there was a clear wish for a thorough introduction to the work at NH by a senior colleague and thereafter recurring meetings with other NH doctors for discussions on elderly care.

*Freedom and variation* was the third category. This is also seen as compared to the work at the PHCC.

*“It is like a relief compared to the PHCC, that you get to go away from the PHCC every week and go to the NH, and it also gives you an opportunity to make your own prioritising”* (GP12)

The GPs felt that the work at the PHCC was rather punctuated and gave no room for flexibility. The work at the NH could be more varied from week-to-week depending on the workload, which created a welcome possibility for shared decision making around their time schedule.

The fourth category was: *Meaningfulness.* The GPs felt that their work at the NH was very important and meaningful.

*“to help people in that situation, it gives me a feeling of having accomplished something good, almost every time I am there”* (GP11)

They felt that they made a difference trying to give the elderly as good a well-being as possible in the latter stages of life. Even if many medications could have exhausted their impact in the elderly, the doctor’s part was to pinpoint where the care needs went beyond medications. The GPs were also satisfied with the fact that they often could prioritise the patients they felt were in most need of care, which gave them opportunities to use their time in the best possible way.

## Discussion

### Principal findings

We found two main themes describing GPs’ experiences of working with elderly in NH: 1. concern for the patient including three categories (*The continuum of ageing, Care needs and medicalisation, View on the nurse as a key person for the well-being of the patient*) and 2. Sustainable working conditions including four categories (*Holism, Collaboration, Freedom and variation and Meaningfulness).*

The main focus for the GPs was the well-being of the patients with special consideration to the continuum of ageing, risk of medicalisation and the need of nursing care. The nurse played many different roles at the NH and an efficient nurse at the NH was extremely important for the GPs ambitions for a good quality of care. An important paradox was presented as the GPs wanted more nursing care and in many cases the nursing staff called for more medications. There is a well-known lack of resources, and maybe an endless need of nursing care for a person with severe dementia, but maybe this could also be seen as all care givers’ (including the GPs’) existential powerlessness when dealing with patients approaching the end of their life. It could be an expression for the feeling that care givers always want to be able to do more for the patients’ well-being, “but it is not on my table”.

The GPs felt satisfied in their work at the NH by having a holistic view of the patients and their task was made manageable with good support from the NH nurse.

### Strengths and weaknesses of the study

Semi-structured interviews with a colleague have both strengths and weaknesses. On one hand there is a mutual respect and understanding of the basics in the profession. On the other hand there can be things left unspoken that are built-in the profession and which cannot be seen from the “outside”. Therefore the co-author with a different profession was chosen to moderate the focus group.

The interviews were conducted in a setting which created a relaxed atmosphere to make the GP comfortable to talk about their experience on a personal level.

In light of the fact of preconceptions of the first author as a GP, who is involved in other NH studies, the co-researchers (also GPs, and one behavioural scientist) viewed the material in every step of the process of analysing data to supplement each other’s statements and interpretations in order to achieve trustworthiness [25].

In this study we used a small sample of GPs from the south of Sweden given that the results are best interpreted in similar settings, but not necessarily transferable to other countries where NH may have a different structure. The interviewed GPs were of differing ages and had diverse types of experience which increased the transferability [22]. Only three of the 12 GPs were male which created a gender imbalance in the group, although in Sweden there is a predominance of females among the younger group of GPs [26]. Working with NH in Sweden is usually optional for a GP. All of the 12 interviewed GPs were interested in elderly care and found their work interesting and important. When participating in an interview study, the GPs had to take time off from the other work at the PHCC. This could lead to a possibility that we may have missed the GPs that did not find the work with elderly interesting and therefore did not want to spend extra time on interviews. This may have been reflected in our study giving a more positive picture of the work at NH. Still, we believe that we have got more information from GPs interested in the subject and wanting it to function well than we would have got from interviewing GPs that were not interested in the task. The focus group was created to deepen the themes derived from the interviews, and maybe to develop new categories. The participating GPs were all satisfied when the categories and themes derived from the interviews were presented. This can be seen as a confirmation of our results which created a thorough member check, strengthening the credibility of our study. The fact that no new categories evolved during the focus group discussion could be because the participants already had said the essence during the interviews, or that the authors were too focused on the pre-existing themes during the focus group discussion.

### Findings compared to other studies and literature

Hospital physicians in Sweden are often frustrated when taking care of elderly NH patients admitted to hospitals because the patients are “too complex and time-consuming to fit in”. The physicians feel they lack the holistic view of the patient and with an inadequate remuneration system and inappropriate routines the care of the elderly patients at hospitals in Sweden is not adapted for elderly with multimorbidity [27]. This needs to be improved but it also shows the importance of stable care at the NH, without inappropriate admissions to the hospital. For optimising the care for the elderly at NH we suggest that the relationship between the GP and the NH nurse is prioritised and it is important to reflect on the fact that a continuous relationship is prosperous.

A study from Norway showed that doctors working in NH were reluctant to dispute nurses’ opinions regarding indications and the effectiveness of drug treatment [28]. It is therefore important to note that the GP is responsible for the decision making on drug treatment based on evidence. There is a lack of professional networks of GPs working with elderly care. The GPs one-by-one set up his or her own way of working with elderly in NH. This is also seen in a recent questionnaire survey, where only 64 % of the GPs involved in nursing homes felt adequately trained for the task [29]. This seems unfruitful and it would be beneficial for the profession to have both professional networks and national guidelines.

The theme Concern for the patient can be interpreted in Lazarus and Folkmann’s [18] perspective on coping strategies in stress. Stress arises when there is an imbalance of resources and demands and their theory describes the *emotion focused coping strategies* and the *problem focused coping strategies* for dealing with stress. The emotion focused coping strategies are that the individual needs to handle and face their own emotions in the situation. Thereafter, the coping strategy can be to create an emotional distance from the situation or try to find a positive glimpse in a hopeless matter. Here, the doctors are careful to note that they are dealing with the continuum of ageing/end of life care. This perspective seems to be well established and in agreement with their own feelings and this makes the decisions around i.e., referring to the hospital or CPR (Cardio Pulmonary Resuscitation) more straight forward.

*“I have done much thinking about how I want to be treated in that situation, and I have talked to my relatives about it. If I get seriously ill and suffer from dementia, please don’t fight for prolonging my life in eternity*” (GP2)

But when it comes to concerns around the patients’ medicalisation and care needs it seems to be harder for the doctors to deal with stress. Problem focused coping strategies means that you try to change a situation to be able to deal with it. Here, the doctors want to change the system. They want more resources, more nursing staff and further education for the nursing staff to solve the problem and minimise their stress in the powerlessness situation. Also the nurse as a key person can help the GP in coping with this stress.

*“I want to get away from giving medicines because of lack of resources in the nursing staff. There is a tendency for this, with too much focus on the doctor.”*(GP11)

The GPs found their NH work enjoyable despite complicated patients, relatives and nursing staff with different demands. This can be seen through Antonovsky’s salutogenic perspective; Sense of Coherence [19]. This is a theoretical formulation for how an individual can deal with stress, describing three components important for the sense of coherence: 1. Comprehensibility, 2. Manageability and 3. Meaningfulness. Comprehensibility refers to a sense that you can understand events in your situation and this can be seen in our category holism. The GPs felt they had a holistic view of the patients which gave them a reliable comprehension of the situation. Manageability is a belief that you have the ability, resources and help necessary to make things manageable and within your control. In our results this is seen in the categories collaboration and freedom and variation, where the GPs described good support from the NH nurse and to some extent also outside resources. They described having control over their time schedule and that they could make their own priorities, thus making their task manageable. Meaningfulness is a sense that there is a good reason or purpose to care about what happens. In the category meaningfulness this was described as very important for the GPs’ satisfaction in that they felt that they made an important difference for the patients in NH.

## Conclusions

The GPs that were interviewed focused on the well-being of the patients and found their work enjoyable and important for the patients at the NH. The perspective on nursing care vs. medications was a recurring subject and there is a need for guidelines and discussions as well as further research on this topic. The striving for the well-being of the patient was eased if there was a good and continuous relationship with the NH nurse.

This study could provide useful information when structuring elderly care in NH, where a continuous GP and nursing staff should be prioritised as well as creating GP networks.

The feeling of satisfaction around the task, such as creating a positive sense of coherence for the GPs, is important to further investigate. The low number of active GPs together with high retirement rates among GPs in Sweden will create problems for future PHC and there is a need for a more solid recruitment base. The GPs in this study present a sense of coherence larger at the NH than at the PHCC. If this could be extrapolated and studied further then this could perhaps provide input to better organise the work for GPs at the PHCC and serve to recruit more doctors to PHC.

## Abbreviations

NH: Nursing homes
GP: General practitioner
PHC: Primary Health Care
PHCC: Primary Health Care Centre

## References

[1] Arai H, Ouchi Y, Yokode M, Ito H, Uematsu H, Eto F, Oshima S, Ota K, Saito Y, Sasaki H (2012). Toward the realization of a better aged society: messages from gerontology and geriatrics. Geriatr Gerontol Int.

[2] Reuben DB, Shekelle PG, Wenger NS (2003). Quality of care for older persons at the dawn of the third millennium. J Am Geriatr Soc.

[3] Katz PR (2011). An international perspective on long term care: focus on nursing homes. J Am Med Directors Assoc.

[4] Tolson D, Rolland Y, Katz PR, Woo J, Morley JE, Vellas B (2013). An international survey of nursing homes. J Am Med Directors Assoc.

[5] National Board of Health and Welfare, Elderly and persons with impairments- management form, March 2013. www.socialstyrelsen.se. Accessed 17 April 2015. [www.socialstyrelsen.se]

[6] Lagergren M (2002). The systems of care for frail elderly persons: the case of Sweden. Aging Clin Exp Res.

[7] Oosterveld-Vlug MG, Pasman HR, van Gennip IE, Willems DL, Onwuteaka-Philipsen BD (2013). Nursing home staff's views on residents' dignity: a qualitative interview study. BMC Health Serv Res.

[8] Modig S, Lannering C, Ostgren CJ, Molstad S, Midlov P (2011). The assessment of renal function in relation to the use of drugs in elderly in nursing homes; a cohort study. BMC Geriatr.

[9] Borgstrom Bolmsjo B, Molstad S, Ostgren CJ, Midlov P (2013). Prevalence and treatment of heart failure in Swedish nursing homes. BMC Geriatr.

[10] Maio V, Jutkowitz E, Herrera K, Abouzaid S, Negri G, Del Canale S (2011). Appropriate medication prescribing in elderly patients: how knowledgeable are primary care physicians? A survey study in Parma, Italy. J Clin Pharm Ther.

[11] Schuling J, Gebben H, Veehof LJ, Haaijer-Ruskamp FM (2012). Deprescribing medication in very elderly patients with multimorbidity: the view of Dutch GPs. A qualitative study. BMC Fam Pract.

[12] Badertscher N, Rossi PO, Rieder A, Herter-Clavel C, Rosemann T, Zoller M (2012). Attitudes, barriers and facilitators for health promotion in the elderly in primary care. A qualitative focus group study. Swiss Medical Weekly.

[13] Groom L, Avery AJ, Boot D, O'Neill C, Thornhill K, Brown K, Jones R (2000). The impact of nursing home patients on general practitioners' workload. Br J Gen Pract.

[14] Jansen K, Schaufel MA, Ruths S (2014). Drug treatment at the end of life: An epidemiologic study in nursing homes. Scand J Prim Health Care.

[15] Jacobs S (2003). Addressing the problems associated with general practitioners' workload in nursing and residential homes: findings from a qualitative study. Br J Gen Pract.

[16] McDermott C, Coppin R, Little P, Leydon G (2012). Hospital admissions from nursing homes: a qualitative study of GP decision making. Br J Gen Pract.

[17] Kirsebom M, Wadensten B, Hedstrom M (2013). Communication and coordination during transition of older persons between nursing homes and hospital still in need of improvement. J Adv Nurs.

[18] Lazarus RS, Folkman S (1984). Stress, Appraisal, and Coping Stress.

[19] Antonovsky A (1978). Unraveling The Mystery of Health -How people manage stress and stay well.

[20] Stein LI (1967). The doctor-nurse game. Arch Gen Psychiatry.

[21] Giorgi A (2005). The phenomenological movement and research in the human sciences. Nurs Sci Q.

[22] Malterud K (2001). Qualitative research: standards, challenges, and guidelines. Lancet.

[23] Crabtree BF Miller W (1999). Doing Qualitative Research, vol. 2.

[24] Kvale S, Brinkmann S (2009). InterViews: learning the craft of qualitative interviewing.

[25] Graneheim UH, Lundman B (2004). Qualitative content analysis in nursing research: concepts, procedures and measures to achieve trustworthiness. Nurse Educ Today.

[26] Socialstyrelsen. Labour Supply in Sweden -Qualified Medical Specialists 2011. In*.* www.socialstyrelsen.se: The National Board of Health and Welfare; 2014.

[27] Ekdahl AW, Hellstrom I, Andersson L, Friedrichsen M: Too complex and time-consuming to fit in! Physicians' experiences of elderly patients and their participation in medical decision making: a grounded theory study. BMJ Open. 2012; 2(3). doi: 10.1136/bmjopen-2012-001063. 10.1136/bmjopen-2012-001063 PMC3367145 22654092

[28] Iden KR, Hjorleifsson S, Ruths S (2011). Treatment decisions on antidepressants in nursing homes: a qualitative study. Scand J Prim Health Care.

[29] Gleeson LE, Jennings S, Gavin R, McConaghy D, Collins DR (2014). Primary care in nursing homes revisited: survey of the experiences of primary care physicians. Ir Med J.

